# Machado: Open source genomics data integration framework

**DOI:** 10.1093/gigascience/giaa097

**Published:** 2020-09-14

**Authors:** Mauricio de Alvarenga Mudadu, Adhemar Zerlotini

**Affiliations:** Embrapa Informática Agropecuária, Campinas, São Paulo, Post Code 13083–886, PO Box 6041, Brazil; Embrapa Informática Agropecuária, Campinas, São Paulo, Post Code 13083–886, PO Box 6041, Brazil

**Keywords:** database, multiomics, Chado, Python

## Abstract

**Background:**

Genome projects and multiomics experiments generate huge volumes of data that must be stored, mined, and transformed into useful knowledge. All this information is supposed to be accessible and, if possible, browsable afterwards. Computational biologists have been dealing with this scenario for more than a decade and have been implementing software and databases to meet this challenge. The GMOD's (Generic Model Organism Database) biological relational database schema, known as Chado, is one of the few successful open source initiatives; it is widely adopted and many software packages are able to connect to it.

**Findings:**

We have been developing an open source software package named Machado, a genomics data integration framework implemented in Python, to enable research groups to both store and visualize genomics data. The framework relies on the Chado database schema and, therefore, should be very intuitive for current developers to adopt it or have it running on top of already existing databases. It has several data-loading tools for genomics and transcriptomics data and also for annotation results from tools such as BLAST, InterproScan, OrthoMCL, and LSTrAP. There is an API to connect to JBrowse, and a web visualization tool is implemented using Django Views and Templates. The Haystack library integrated with the ElasticSearch engine was used to implement a Google-like search, i.e., single auto-complete search box that provides fast results and filters.

**Conclusion:**

Machado aims to be a modern object-relational framework that uses the latest Python libraries to produce an effective open source resource for genomics research.

## Introduction

The technological advances for biological research regarding genomic sequencing, phenotype prediction, and re-engineering of living systems have led to the creation of large volumes of data that must be stored, mined, and transformed into useful knowledge. These technological advances of the omic approaches, including genomics, transcriptomics, proteomics, and metabolomics, have great impact in several areas of knowledge including agriculture, especially for integrating big data analysis into animal and plant breeding programs.

Omics enables a systems biology approach toward understanding the complex interactions between genes, proteins, and metabolites within the resulting phenotype [1]. Omics data integration offers the potential to increase the productivity and sustainability of crop and livestock production. The challenges are diverse but usually consist of identifying genetic variation that derives desirable traits that can drive genomic prediction, performing precise genome editing/engineering (e.g., using CRISPR-CAS systems for the induction of mutations or disruptions in the genome), identifying molecular targets for developing vaccines to diseases/plagues, and probably others [2].

All this novel genomic information, especially those from genome projects and multiomics experiments (e.g., transcriptomics, proteomics), is supposed to be accessible and, if possible, browsable afterwards. Furthermore, a great challenge is to integrate data from different organisms and projects for analysis and mining of data. Plant and animal trait data are typically generated by diverse, costly, and time-consuming experiments and thus can hugely benefit from increased data sharing and integration [3]. Bioinformaticians and computational biologists have been dealing with this scenario for more than a decade now and have implemented (and are still implementing) a collection of software libraries, toolkits, platforms, databases, and data warehouses in this regard.

Although public databases exist, research groups still struggle to store and analyse data with local resources and expertise. The Generic Model Organism Database project (GMOD) is currently the initiative that is the most advanced in producing a “collection of open source software tools for managing, visualizing, storing and disseminating genomic data” [4]. Its biological relational database schema named Chado [5] is widely adopted, and many software packages are able connect to it, e.g., Jbrowse [6], Gbrowse [7], Apollo [8], Intermine [9], and Tripal [10].

The development of a front end for Chado named Tripal, based on the Drupal content management system, facilitated the construction and publication of genomic databases [11], although historically PHP is barely used in bioinformatics. For instance, the latest release of BioPHP, a collection of open source PHP code with a number of bioinformatics tools, is from 2003 [12].

It can be argued that Python has become the de facto standard for exploratory, interactive, and computation-driven scientific research [13]. Ranking first in the top programming languages of 2019 [14], Python has a vast collection of libraries and modules for bioinformatics, e.g., BioPython [15], PyVCF, and PySAM; and data science, e.g., SciPy, NumPy, pandas, and Matplotlib. Coupled with Django, one of the most mature and feature-rich web application frameworks for Python [16], it can be used to produce fast, secure, and scalable software.

The Embrapa Bioinformatics Multi-user Laboratory began to develop an open source software package called Machado that has a Django model to connect to Chado, thus obviating extra efforts to make data compatible to the database schema.

The Chado database schema enables us to integrate different data types using controlled vocabularies and ontologies. For example, the Sequence Ontology [17], a collection of sequence feature types, is used for typing features in the sequence module of Chado. Therefore, every biological component and its relationships are formally described, allowing the identification of exons, transcripts and other features that are part of a gene. Additionally, the Gene Ontology [18] enables us to functionally characterize the biological components in terms of their molecular function, cellular localization, and the biological process with which they are involved.

The Django framework provides the practical means to build visualization tools and APIs to assist software developers in dealing with multiple genomic data sources for building seamless, interoperable applications. The API framework is a set of clearly defined methods of communication between various software components. Data standardization across different research groups, coupled with the API framework, will facilitate future collaborations with data scientists to explore the data sets even further.

We intend to provide a Python framework to store diverse biological data, make complex queries, and visualize results for this project. Furthermore, we hope to broaden the usability of the Machado software and provide the community with a powerful, simple, open source software tool that could be used by other scientific groups in projects of a range of complexities.

## Findings

Machado is a Django instance that provides data management, visualization, and search functionalities to Chado databases. The resulting object-relational framework enables users not only to set up a local instance containing data regarding their organisms of interest but also to develop all sorts of tools by accessing the open source code.

The data-loading tools are currently available via the Django management interface. Such tools were developed to load data from the most common bioinformatics file formats to the Chado database. This implementation provides users with commands capable of loading data files using multi-thread and real-time progress monitoring. Developers are welcome to create or propose new data formats to be implemented in future versions.

Machado also provides users with an out-of-the-box solution to browse the data, which is able to display every datum loaded using the current data-loading tools. This web interface is fully customizable to encourage the development of new solutions or connection to analysis tools.

**Figure 1:**
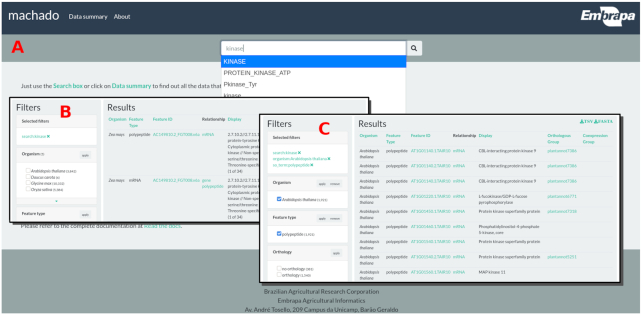
(A) The Machado demonstration site home page, the search form, and autocomplete component; (B) the search results for the term “kinase”; (C) the search results for the term “kinase,” filtered by organism “*Arabidopsis thaliana*” and feature type “polypeptide”.

The current version can be tested at a demonstration website [19], which contains genomics data from 5 plants. This interface aims to simplify data searching by providing users with a single search box to query all the data. The user would open the web page and type a given term (Fig. 1A) to gain instant access to similar valid keywords provided by the autocomplete feature. After typing the keywords and submitting the form, the user is redirected to the search results page (Fig. 1B). This page contains summarized information about genes, transcripts, and proteins from all the organisms and filtering boxes in the lefthand section that enable the selection of features by specific criteria, such as organism, type, orthology, coexpression, and annotation. It also provides column sorting, control of the number of results, and download of the table or the sequences (Fig. 1C).

**Figure 2:**
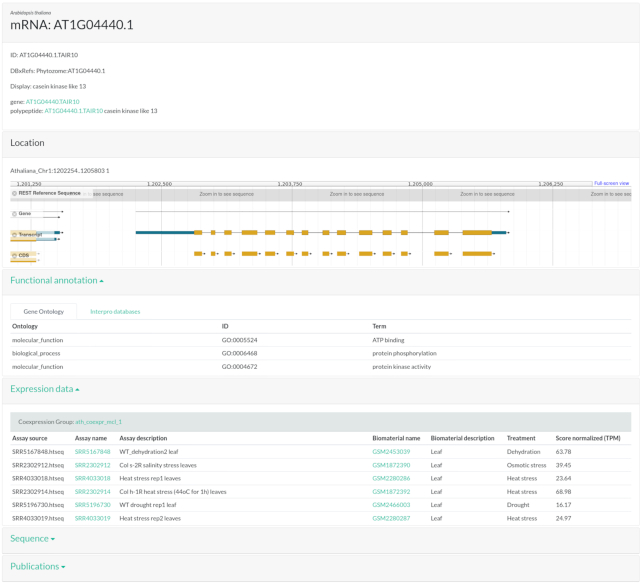
Feature page.

The search results columns contain hyperlinks to the feature itself (e.g., protein) and its related features (e.g., messenger RNA [mRNA]), orthologous groups, or coexpression groups. By clicking on a hyperlink of a feature the user is redirected to the feature page that contains detailed information about it (Fig. 2).

The feature page is organized in cards that can be collapsed to facilitate the visualization of specific information. The first card contains information about the feature itself, such as the organism, IDs, related features, and text annotation. If the feature is mapped to the genome, the next card will show the genomic location and an embedded genome browser powered by JBrowser. The following cards contain detailed information about functional annotation, ortholog groups, coexpression groups, sequence, and publications.

## Methods

### The database model

The Machado software was developed and tested using Python 3 and Django 2.2.10. The first step was to create an object-relational model for the GMOD Chado database schema 1.31, using the Django inspectdb tool and a custom Python script that is available within the Machado repository. The resulting model integrates the Machado package and is used solely to connect to a Chado database, not to create the tables. During a new Machado installation, the original GMOD Chado schema SQL file is used to create the database instance. Therefore, users should be able to provide an already populated database instance if desired.

### The data-loading tools

The data-loading tools were developed following the interface segregation principle, according to which, the classes related to ETL (extract, transform, and load) are independent from the classes related to the user interface. This design pattern makes it possible to implement diverse user interfaces (command line, web, or API) while preserving the ETL classes.

The user interface was implemented using the Django management tool, a command line management system to execute database-related tasks in a standard manner. The management tool, together with a few Python libraries, allowed us to implement an effective set of data-loading commands capable of loading data files using multi-threading and providing users with real-time progress monitoring.

The ETL classes benefit from well-established bioinformatics libraries to handle the genomics data files, namely, BioPython to load FASTA, GFF, BLAST, and InterproScan; obonet to load ontology files; and bibtexparser to load Bibtex files.

### The web interface

The Machado web interface was developed using the Django views and templates, which is a convenient way to generate HTML dynamically. The core set of web pages consists of a search form, a search results page, and a feature page.

The search form is embedded in the page header to allow users to conveniently search for features at any time. The search form contains autocomplete capabilities to help users to validate keywords, i.e., to identify keywords that are stored in the database and verify their correct spelling.

Once a query is executed, the search form redirects the web browser to the results page, which contains basic information on every feature that meets the search criteria. The search results are paginated, and this page also provides filtering, text download, sequence download, column sorting, and selection of the number of records per page.

Every feature has a hyperlink that leads to the feature page, which contains every piece of information stored in the database related to the feature itself, e.g., genes, transcripts, and proteins. Currently, it is set to show ID, description, relationships, genomic location, functional annotation, similarity analysis results, co-expression networks, groups of orthologs, expression data, sequence, and related publications. The genomic location is rendered with an embedded JBrowse instance to enhance the user's experience. It shows the genomic region in detail, the gene structure with its exons, and untranslated regions, and allows the user to zoom in or out to identify adjacent genes.

### The search engine

The Machado search and filter components are powered by the ElasticSearch engine that is invoked via the Haystack library. This approach allowed us to create a single search box that autocompletes the user keywords and finds matches throughout the whole database. The search results can be filtered by organism, type, orthology, coexpression, annotation, expression biomaterial, and expression treatment. This filtering component was implemented using the ElasticSearch faceted navigation, which allows users to sort vast data sets by applying filters.

Both the search and the indexing components are invoked via the Haystack library, which significantly simplified the software programming process because it uses familiar Django syntax rather than native ElasticSearch coding. The Haystack library can directly connect to the Django model and retrieve data to a few search engines, such as ElasticSearch and Solr. The Machado repository contains the specific code to generate the search index based on the Chado schema. Therefore, after having all the data loaded up, the user will simply build the search index using a specific command in the Django management tool.

## Discussion

Although Machado aims to simplify genomics data integration, it requires considerable understanding of the data that are being loaded as well as of the computational tools. The GMOD Chado database schema relies upon ontologies to establish the relationship among data types, and therefore the data must be loaded in a particular order to ensure that the parent data are always loaded in advance. The user must observe the file format specifications and ascertain that the feature IDs are consistent throughout the files. In regard to the computational tools, it is necessary to carry out systems administration tasks related to software installation and configuration, user permissions, and evaluation of hardware requirements. There are other frameworks to integrate genomic data such as Intermine or Tripal that are in more advanced stages of development, but nevertheless users will have to perform the same aforementioned laborious tasks.

The development of Machado was proven to be very fortunate once we started taking advantage of the Python libraries. For instance, Biopython enabled us to parse several file formats effectively and Haystack enabled very fast search and filtering capabilities. The single search box with autocomplete and faceting capabilities is arguably unprecedented among the open source frameworks available. The Python library repository is vast, and therefore there is much to expand on future releases. For example, Machado can be used not only to host genomics data but also to enable the development of specific tools, such as PlantAnnot [20], to identify and annotate genes of interest. There is extensive documentation within the Machado repository, and users are welcome to contact us and propose documentation updates.

## Conclusion

The Machado software is a modern open source Python framework constructed to store, integrate, query, and visualize multiomics data and also to be fast and easy to use. The software is public and everyone can download, use, and collaborate by proposing improvements and submitting code.

## Availability of Supporting Source Code and Requirements

Project name: Machado

- Project home page: https://github.com/lmb-embrapa/machado
- Operating system(s): Platform independent
- Programming language: Python
- Other requirements: Python 3 or higher, PostgreSQL 10 or higher, and ElasticSearch 5
- License: GNU GPL 3
- RRID:SCR_018428
- biotools: machado

The complete source code is available in GitHub and the tests are executed routinely, triggered by Travis-CI for every new code commit. Extensive testing and code reviews ensure that the software is fully functional upon new installations. Detailed instructions hosted at Read The Docs [21] describe how to install, load data, and set up the user interface. The GitHub repository also hosts the Docker image and instructions on how to create a Docker instance of Machado [22].

## Availability of Supporting Data and Materials

The data sets supporting the results of this article are available in the Phytozome V12 repository [23], Athaliana_167_TAIR10, Dcarota_388_v2.0, Dcarota_388_v2.0, Phytozome: Dcarota_388_v2.0, and Zmays_284_Ensembl-18_2010–01-MaizeSequence. Snapshots of our code, Docker image, and other supporting documentation and data can be openly found in the *GigaScience* repository, GigaDB [24].

## Abbreviations

API: Application Programming Interface; BLAST: Basic Local Alignment Search Tool; CRISPR: clustered regularly interspaced short palindromic repeats; ETL: extract, transform, and load; GMOD: Generic Model Organism Database.

## Competing Interests

The authors declare that they have no competing interests.

## Funding

This work was supported by Empresa Brasileira de Pesquisa Agropecuária—Embrapa 13.16.04.010.00.00–PLANTANNOT—Implementation of a bioinformatics pipeline for gene discovery related to abiotic stresses in plants—principal Investigator: Mauricio de Alvarenga Mudadu.

## Authors' Contributions

Both authors conceived the project; A.Z. implemented most of the code and tested the source code; M.M. helped implement the data-loading code and tested the source code; both authors drafted the manuscript, provided final editing, and read and approved the final manuscript.

## Supplementary Material

giaa097_GIGA-D-20-00137_Original_Submission

giaa097_GIGA-D-20-00137_Revision_1

giaa097_GIGA-D-20-00137_Revision_2

giaa097_GIGA-D-20-00137_Revision_3

giaa097_Response_to_Reviewer_Comments_Original_Submission

giaa097_Response_to_Reviewer_Comments_Revision_1

giaa097_Response_to_Reviewer_Comments_Revision_2

giaa097_Reviewer_2_Report_Original_Submission Daniel Cook -- 6/28/2020 Reviewed
