# Supplementary material for: Machado: Open source genomics data integration framework
Source: Gigascience. 2020 Sep 14;9(9):giaa097. doi: 10.1093/gigascience/giaa097 (PMC7490629; doi:10.1093/gigascience/giaa097)

GigaScience

Machado: open source genomics data integration framework

--Manuscript Draft--

**Manuscript Number:**	GIGA-D-20-00137R3
**Full Title:**	Machado: open source genomics data integration framework
**Article Type:**	Technical Note
**Funding Information:**	EMBRAPA (13.16.04.010.00.00 - PLANTANNOT - Implementation of a bioinformatics pipeline for gene discovery related to abiotic stresses in plants) — Dr., Mauricio de Alvarenga Mudadu
**Abstract:**	Background<br>Genome projects and multiomics experiments generate huge volumes of data that must be stored, mined and transformed into useful knowledge. All this information is supposed to be accessible and, if possible, browsable afterwards. Computational biologists have been dealing with this scenario for over a decade and have been implementing softwares and databases to succeed in this matter. The GMOD's (Generic Model Organism Database) biological relational database schema, known as Chado, is one of the few successful open source initiatives, it is widely adopted and many softwares are able to connect to it. Findings<br>We have been developing an open source software named Machado, a genomics data integration framework implemented in Python, to enable research groups to both store and visualize genomics data. The framework relies on the Chado database schema and, therefore, should be very intuitive for current developers to adopt it or have it running on the top of already existing databases. It has several data loading tools for genomics and transcriptomics data and also for annotation results from tools such as BLAST, InterproScan, OrthoMCL and LSTrAP. There is an API to connect to JBrowse and a web visualisation tool is implemented using Django Views and Templates. The Haystack library integrated with the ElasticSearch engine was used to implement a google-like search i.e. single auto-complete search box that provides fast results and filters. Conclusion<br>Machado aims to be a modern object-relational framework that uses the latests Python libraries to produce an effective open source resource for genomics research.
**Corresponding Author:**	Adhemar Zerlotini<br>Embrapa Informatica Agropecuaria<br>BRAZIL
**Corresponding Author Secondary Information:**	
**Corresponding Author's Institution:**	Embrapa Informatica Agropecuaria
**Corresponding Author's Secondary Institution:**	
**First Author:**	Mauricio de Alvarenga Mudadu, Ph.D.
**First Author Secondary Information:**	
**Order of Authors:**	Mauricio de Alvarenga Mudadu, Ph.D.
	Adhemar Zerlotini
**Order of Authors Secondary Information:**	
**Response to Reviewers:**	Editor<br><br>The manuscript was reformatted to conform with the Instructions for Authors for Technical Notes. It was submited as an word file and the figures were attached as well. Thanks for the comments, each one of them was addressed in order to conform to the instructions.

Powered by Editorial Manager® and ProduXion Manager® from Aries Systems Corporation

Reviewer 1

1) How can new analysis tools be integrated in this framework? Multi-omics data analysis is a more critical issue than data storing or visualization. What kinds of data analytics tool will be available? How to provide the flexibility to integrate third-party software in this framework?

Currently, there are several multi-omics data sets being generated in our institution and it's a major issue Machado intended to deal with primarily. We decided that any multi-omics data analysis should initially be processed separately and the results loaded to the database. This enables users to select features based on the analysis results without having to wait for the analysis to run, which makes Machado faster and simpler. However, this can change in the future. It it possible to integrate analysis tools to Machado as it provides direct access to features, sequences and analysis results via API. There's much to improve in the API interface and we're working on it regularly. Developers can also access the data via the object-relational model or the ElasticSearch index.

2) For the data integration, how can external database be integrated? The framework provides the flexibility to link with other databases? Databases does not only mean relational databases, but can be multiple data sources or biological databases such as KEGG.

The current version of Machado already contains links to other databases such as the NCBI SRA and GEO databases. It's quite simple to add links to external databases by editing the feature template. Each feature may have several dbxrefs (DB external reference) that enable to directly link to external data sources. The integration with different data sources, such as KEGG, is not complicated. Our objective is really to expand these data sources in order to integrate as much information as possible.

3) What kinds of benefit does the function of search powered by ElasticSearch engine provide to users? Seems all components already exist and this proposed framework just implemented by integrating them.

The search engines available in other open source frameworks to integrate multi-omics data are split in several search boxes for different parameters. For example: ID search, GO term search, Gene name search, Organism search, and so on. The user have to execute each search separately and then run another tool to intersect the searches results. The benefit of using the ElasticSearch is to execute complex queries using a single search box, such as, 'calcium GO:0016020 PF02705', which will select features that transport (PF02705) calcium through the membrane (GO:0016020). The result of this query can be additionally filtered by several criteria, such as, organism, expression, orthology, and so on. Although it's true that "components already exist and the framework just implemented it", the novelty is that Machado provides the software to generate and access the ElasticSearch index automatically from a Chado database.

Reviewer 2

- The export icons for TSV/FASTA are identical. This is a UX issue that should be addressed.

The export icons were updated.

- To facilitate deployment/usage, it may be beneficial to create a Dockerfile that can read a configuration and allow users to immediately test Machado on their existing Chado database.
The docker image for Machado is available at https://github.com/lmb-

	embrapa/machado-docker<br>This repository also contains a docker-composer and sample data in order to have a Machado instance running straightway.<br><br>- documentation for the API appears to be lacking. When I go to https://www.machado.cnptia.embrapa.br/demo_machado/api/ I get 'undefined' for some queries, and it is unclear how to use the API.<br><br>The Machado API was completely refactored. Now it provides OpenAPI support (https://www.machado.cnptia.embrapa.br/demo_machado/api/?format=openapi) and Swagger documentation (https://www.machado.cnptia.embrapa.br/demo_machado/api/).<br><br>Within the manuscript, there were some minor wording issues the authors should consider revising. For example, it is unclear what a "public wide database" is. There are also a handful of grammatical errors in the manuscript that need to be fixed. Also, the first github link appears to be broken.<br><br>The manuscript was revised for grammatical errors. The link was fixed.
**Additional Information:**	
**Question**	**Response**
Are you submitting this manuscript to a special series or article collection?	No
**Experimental design and statistics**<br><br>Full details of the experimental design and statistical methods used should be given in the Methods section, as detailed in our Minimum Standards Reporting Checklist. Information essential to interpreting the data presented should be made available in the figure legends.<br><br>Have you included all the information requested in your manuscript?	Yes
**Resources**<br><br>A description of all resources used, including antibodies, cell lines, animals and software tools, with enough information to allow them to be uniquely identified, should be included in the Methods section. Authors are strongly encouraged to cite Research Resource Identifiers (RRIDs) for antibodies, model organisms and tools, where possible.<br><br>Have you included the information requested as detailed in our Minimum	Yes

Standards Reporting Checklist?	
**Availability of data and materials**<br><br>All datasets and code on which the conclusions of the paper rely must be either included in your submission or deposited in publicly available repositories (where available and ethically appropriate), referencing such data using a unique identifier in the references and in the “Availability of Data and Materials” section of your manuscript.<br><br>Have you have met the above requirement as detailed in our Minimum Standards Reporting Checklist?	Yes

Powered by Editorial Manager® and ProduXion Manager® from Aries Systems Corporation

Manuscript with DOI

Click here to access/download;Manuscript;ArtigoMachado_rev_v7 with

Machado: open source genomics data integration framework

Mauricio de Alvarenga Mudadu[1], Adhemar Zerlotini[1,*],

[1] Embrapa Informática Agropecuária, Campinas, São Paulo, Post Code 13083-886, PO box 6041, Brazil

* To whom correspondence should be addressed. Tel: +55 19 32115700; Fax: +55 19 3211-5754; Email:adhemar.zerlotini@embrapa.br

Mauricio de Alvarenga Mudadu <mauricio.mudadu@embrapa.br>
Adhemar Zerlotini <adhemar.zerlotini@embrapa.br>

Abstract

Background

Genome projects and multiomics experiments generate huge volumes of data that must be stored, mined and transformed into useful knowledge. All this information is supposed to be accessible and, if possible, browsable afterwards. Computational biologists have been dealing with this scenario for over a decade and have been implementing softwares and databases to succeed in this matter. The GMOD's (Generic Model Organism Database) biological relational database schema, known as *Chado*, is one of the few successful open source initiatives, it is widely adopted and many softwares are able to connect to it.

Findings

We have been developing an open source software named *Machado*, a genomics data integration framework implemented in *Python*, to enable research groups to both store and visualize genomics data. The framework relies on the *Chado* database schema and, therefore, should be very intuitive for current developers to adopt it or have it running on the top of already existing databases. It has several data loading tools for genomics and transcriptomics data and also for annotation results from tools such as *BLAST*, *InterproScan*, *OrthoMCL* and *LSTrAP*. There is an API to connect to *JBrowse* and a web visualisation tool is implemented using *Django Views and Templates*. The *Haystack* library integrated with the *ElasticSearch* engine was used to implement a google-like search i.e. single auto-complete search box that provides fast results and filters.

Conclusion

Machado aims to be a modern object-relational framework that uses the latests *Python* libraries to produce an effective open source resource for genomics research.

Keywords: database, multiomics, Chado, python

Introduction

The technological advances for biological research regarding genomic sequencing, phenotype prediction and re-engineering of living systems have led to the creation of large volumes of data that must be stored, mined and transformed into useful knowledge. These technological advances of the omic approaches, including genomics, transcriptomics, proteomics and metabolomics, have great impact in several areas of knowledge including agriculture, specially for integrating big data analysis into animal and plant breeding programs.

Omics enables a systems biology approach toward understanding the complex interactions between genes, proteins, and metabolites within the resulting phenotype [1]. Omics data integration offers the potential to increase the productivity and sustainability in crop and livestock production. The challenges are diverse but are usually composed of identifying genetic variation that derive desirable traits that can drive genomic prediction, performing precise genome editing/engineering (e.g.: using CRISPR-CAS systems for the induction of mutations or disruptions in the genome), identifying molecular targets for developing vaccines to diseases/plagues, and probably others [2].

All these novel genomic information, specially those from genome projects and multiomics experiments (transcriptomics, proteomics, etc.) is supposed to be accessible and, if possible, browseable afterwards. Even further, a great challenge is to integrate data from different organisms and projects for analysis and mining of data. Plant and animal trait data are typically generated by diverse, costly and time-consuming experiments, and thus can hugely benefit from increased data sharing and integration [3]. Bioinformaticians and computational biologists have been dealing with this scenario for over a decade now and have implemented (and are still implementing) a collection of software libraries, toolkits, platforms, databases and data warehouses in this regard.

Although public databases exist, research groups still struggle to store and analyse data with local resources and expertise. The GMOD, Generic Model Organism Database project, is currently the initiative that most advanced in producing a "collection of open source software tools for managing, visualizing, storing and disseminating genomic data" [4] . Its biological relational database schema named Chado [5] is widely adopted and many softwares are able connect to it eg. Jbrowse [6] , Gbrowse [7] , Apollo [8] , Intermine [9] , and Tripal [10] .

The development of a front-end for Chado named Tripal, based on the Drupal CMS, facilitated the construction and publication of genomic databases [11] , although historically PHP is barely used in bioinformatics. For instance, BioPHP, a collection of open source PHP code with a number of bioinformatics tools latest release is from 2003 [12] .

“Python has arguably become the de facto standard for exploratory, interactive, and computation-driven scientific research” [13]. Ranking first in the 2019 IEEE Spectrum top programming languages [14] , Python has a vast collection of libraries and modules for bioinformatics eg. BioPython [15] , PyVCF, and PySAM; and data science eg. SciPy, NumPy, pandas, and Matplotlib. Coupled with Django, one of the most mature and feature-rich web application framework for Python [16] , developers are able to produce fast, secure, and scalable softwares.

The Embrapa's Bioinformatics Multi-user Laboratory began to develop an open source software called Machado that has a Django Model to connect to Chado, thus avoiding extra efforts to make data compatible to the database schema.

The Chado database schema enables us to integrate different data types using controlled vocabularies and ontologies. For example, the Sequence Ontology [17], a collection of sequence feature types, is used for typing features in the sequence module of Chado. Therefore, every biological component and its relationships are formally described, allowing the identification of exons that are 'part_of' a gene or transcripts that are 'part_of' a gene. Additionally, the Gene Ontology [18] enables us to functionally characterize the biological components regarding to their molecular function, cellular localization and the biological process they are involved with.

The Django framework provides practical means to build visualization tools and APIs (Application Programming Interface) to assist software developers to deal with multiple genomic data sources for building seamless, interoperable applications. The API framework is a set of clearly defined methods of communication between various software components. The data standardization across different research groups, coupled with the API framework, will facilitate future collaborations with data scientists in order to explore the data sets even further.

We intend to provide a Python framework to store diverse biological data, make complex queries and visualize results for this project. But even further, we hope to broaden the Machado software usability and provide the community with a powerful, simple, open source software that could be used by other scientific groups in projects of many ranges of complexities.

Findings

Machado is a Django instance that provides data management, visualization, and searching functionalities to Chado databases. The resulting object-relational framework enables users, not only to set up a local instance containing data regarding their organisms of interest, but also to develop all sorts of tools by accessing the open source code.

Figure 1: A) the Machado demonstration site home page, the search form, and autocomplete component; B) the search results for the term 'kinase'; C) the search results for the term 'kinase', filtered by organism '*Arabidopsis thaliana*', and and feature type 'polypeptide'.

The data loading tools are currently available via the Django management interface. Such tools were developed to load data from the most common bioinformatics file formats to the Chado database. This implementation provides users with commands capable of loading data files using multi-thread and real-time progress monitoring. Developers are welcome to create or propose new data formats to future versions.

Machado also provides users with an out-of-the-box solution to browse the data, which is able to display every data loaded using the current data loading tools. This web interface is fully customizable in order to encourage the development of new solutions or the connection to analysis tools.
The current version can be tested at a demonstration website [19] which contains genomics data from five plants. This interface aims to simplify data searching by providing users with a single search box to query all the data. The user would open the web page and type a given term (Figure 1-A) to have instant access to similar valid keywords provided by the autocomplete feature. After typing the keywords and submitting the form, the user is redirected to the search results page (Figure 1-B). Such page contains summarized information about genes, transcripts, and proteins from all the organisms and filtering boxes at the left section which enables selecting features by specific criteria, such as organism, type, orthology, coexpression, and annotation. It also provides column sorting, control of the number of results, and download of the table or the sequences (Figure 1-C).

The search results columns contain hyperlinks to the feature itself (eg. protein), its related feature (eg. mRNA), orthologous groups, or coexpression groups. By clicking in a Hyperlink of a feature the user is redirected to the feature page that contains detailed information about it (Figure 2).

Figure 2: Feature page

The feature page is organized in cards that can be collapsed to facilitate the visualization of specific information. The first card contains information about the feature itself, such as the organism, IDs, related features, and text annotation. If the feature is mapped to the genome, the next card will show the genomic location and an embedded genome browser powered by JBrowser. The following cards contain detailed information about functional annotation, orthologs groups, coexpression groups, sequence, and publications.

Methods

The database model

The *Machado* software was developed and tested using Python3 and Django 2.2.10. The first step was to create an object-relational model for the GMOD Chado database schema 1.31, using the Django inspectdb tool and a custom Python script that is available within the Machado repository. The resulting model integrates the Machado package and is used solely to connect to a Chado database, not to create the tables. During a new Machado installation, the original GMOD Chado schema SQL file is used to create the database instance. Therefore, users should be able to provide an already populated database instance if desired.

The data loading tools

The data loading tools were developed following the interface segregation principle, in which, the classes related for ETL (extract, transform, and load) are independent from the classes related to the

user interface. This design pattern makes it possible to implement diverse user interfaces (command-line, web, or API) while preserving the ETL classes.
The user interface was implemented using the Django management tool, a command-line management system to execute database related tasks in a standard manner. The management tool, together with a few Python libraries, allowed us to implement an effective set of data loading commands capable of loading data files using multi-threading and providing users with real-time progress monitoring.
The ETL classes benefit from well-established bioinformatics libraries to handle the genomics data files, namely, BioPython to load FASTA, GFF, Blast, and InterproScan; obonet to load ontology files; and bibtexparser to load Bibtex files.

The web interface

The Machado web interface was developed using the Django views and templates, which is a convenient way to generate HTML dynamically. The core set of web pages is constituted of a search form, a search results page, and a feature page.
The search form is embedded in the page header in order to allow users to conveniently search for features at any time. The search form contains autocomplete capabilities to help users to validate keywords i.e. to identify keywords that are stored in the database and its correct spelling.
Once a query is executed, the search form redirects the web browser to the results page, which contains basic information of every feature that meets the search criteria. The search results are paginated and this page also provides filtering, text download, sequence download, column sorting, and selection of the number of records per page.
Every feature has an hyperlink that leads to the feature page, which contains every information stored in the database related to the feature itself eg. genes, transcripts, and proteins. Currently, it's set to show ID, description, relationships, genomic location, functional annotation, similarity analysis results, co-expression networks, groups of orthologs, expression data, sequence, and related publications. The genomic location is rendered with an embedded JBrowse instance in order to enhance the users experience. It shows the genomic region in detail, the gene structure with its exons, and UTRs, and allows to zoom in/out to identify adjacent genes.

The search engine

The Machado search and filter components are powered by the ElasticSearch engine that is invoked via the Haystack library. This approach allowed us to create a single search box that autocompletes the user keywords and finds matches throughout the whole database. The search results can be filtered by organism, type, orthology, coexpression, annotation, expression biomaterial, and expression treatment. This filtering component was implemented using the ElasticSearch faceted navigation which allow users to filter through vast data sets by checking filters.
Both the search and the indexing components are invoked via the Haystack library, which significantly simplified the software programming process since it uses familiar Django syntax, rather than native ElasticSearch coding. The Haystack library is able to directly connect to the Django model and

retrieve data to a few search engines, such as, ElasticSearch, and Solr. The Machado repository contains the specific code to generate the search index based on the Chado schema. Therefore, after having all the data loaded up, the user will simply build the search index using a specific command in the Django management tool.

Discussion

Although Machado aims to simplify the genomics data integration, it requires considerable understanding of the data that's being loaded as well as of the computational tools. The GMOD Chado database schema relies upon ontologies to establish the relationship among data types and, therefore, the data must be loaded in a particular order to ensure the parent data is always loaded in advance. The user must observe the file format specifications and ascertain the feature IDs are consistent throughout the files. In regard to the computational tools, it's required to carry out systems administration tasks related to software installation and configuration, users permissions, and hardware requirements evaluation. There are other frameworks to integrate genomic data such as Intermine or Tripal that are in more advanced stages of development, but nevertheless users will have to go through the same laborious tasks described above.

The development of Machado was proven to be very fortunate once we started taking advantage of the Python libraries. For instance, Biopython enabled us to parse several file formats effectively and Haystack enabled very fast search and filtering capabilities. The single search box with autocomplete and faceting capabilities is arguably unprecedented among the open source frameworks available. The Python library repository is vast and, therefore, there is much to expand on future releases. For example, Machado can be used not to only host genomics data but also to enable the development of specific tools, such as, PlantAnnot [20] , to identify and annotate genes of interest .There's extensive documentation within the Machado repository and users are welcome to contact us and propose documentation updates.

Conclusion

The Machado software is a modern open source python framework that intends to store, integrate, query, and visualize multiomics data and also to be fast and easy to use. The software is public and everyone can download, use and collaborate by proposing improvements and submitting code.

Availability of supporting source code and requirements

Project name: Machado

- Project home page: https://github.com/lmb-embrapa/machado
- Operating system(s): Platform independent
- Programming language: Python
- Other requirements: Python 3 or higher, PostgreSQL 10 or higher, and ElasticSearch 5
- License: GNU GPL 3
- RRID:SCR_018428

- biotools:machado

The complete source code is available in GitHub and the tests are executed routinely, triggered by Travis-CI for every new code commit. Extensive testing and code reviews ensure the software is fully functional upon new installations. There's also detailed instructions hosted at Read The Docs [21] on how to install, load data, and set up the user interface. The GitHub repository also hosts the Docker image and instructions on how to create a Docker instance of Machado [22] .

Availability of supporting data

The data sets supporting the results of this article are available in the Phytozome V12 repository [23] ,
Athaliana_167_TAIR10, Dcarota_388_v2.0, Dcarota_388_v2.0, Phytozome: Dcarota_388_v2.0, and Zmays_284_Ensembl-18_2010-01-MaizeSequence.
Snapshots of our code, Docker image, and other supporting documentation and data, can be openly found in the *GigaScience* repository, GigaDB [24].

Competing interests

The authors declare that they have no competing interests.

Funding

Empresa Brasileira de Pesquisa Agropecuária - Embrapa 13.16.04.010.00.00 – PLANTANNOT – Implementation of a bioinformatics pipeline for gene discovery related to abiotic stresses in plants – Principal Investigator: Mauricio de Alvarenga Mudadu

Acknowledgements

Many thanks for Embrapa Multiuser Bioinformatics Laboratory (LMB - Laboratório Multiusuário de Bioinformática da Embrapa) and Embrapa Agricultural Informatics (Embrapa Informática Agropecuária) for all the support.

Authors contributions

MM and AZ conceived the project; AZ implemented most of the code and tested the source code; MM helped implementing the data loading code and tested the source code; MM and AZ drafted the manuscript and provided final editing. All authors read and approved the final manuscript.

References

1. Van Emon JM. The Omics Revolution in Agricultural Research. *J Agric Food Chem*. 2016; doi: 10.1021/acs.jafc.5b04515.

2. Huang S, Chaudhary K, Garmire LX. More Is Better: Recent Progress in Multi-Omics Data Integration Methods. *Front Genet*. 2017; doi: 10.3389/fgene.2017.00084.

3. Leonelli S, Davey RP, Arnaud E, Parry G, Bastow R. Data management and best practice for plant science. *Nat Plants*. 2017; doi: 10.1038/nplants.2017.86.

4. GMOD: Chado Manual - GMOD. gmod.org (2020). Accessed 10 Aug 2020.

5. Mungall CJ, Emmert DB. A Chado case study: an ontology-based modular schema for representing genome-associated biological information. *Bioinformatics*. 2007; doi: 10.1093/bioinformatics/btm189.

6. Skinner ME, Uzilov A V., Stein LD, Mungall CJ, Holmes IH. JBrowse: A next-generation genome browser. *Genome Res*. 2009; doi: 10.1101/gr.094607.109.

7. Stein LD, Mungall C, Shu S, Caudy M, Mangone M, Day A, et al.. The Generic Genome Browser: A Building Block for a Model Organism System Database. *Genome Res*. 2002; doi: 10.1101/gr.403602.12.

8. Lee E, Helt GA, Reese JT, Munoz-Torres MC, Childers CP, Buels RM, et al.. Web Apollo: a web-based genomic annotation editing platform. *Genome Biol*. 2013; doi: 10.1186/gb-2013-14-8-r93.

9. Kalderimis A, Lyne R, Butano D, Contrino S, Lyne M, Heimbach J, et al.. InterMine: extensive web services for modern biology. *Nucleic Acids Res*. 2014; doi: 10.1093/nar/gku301.

10. Spoor S, Cheng C-H, Sanderson L-A, Condon B, Almsaeed A, Chen M, et al.. Tripal v3: an ontology-based toolkit for construction of FAIR biological community databases. *Database*. 2019; doi: 10.1093/database/baz077.

11. Tripal: Tripal. http://tripal.info (2020). Accessed 10 Aug 2020.

12. BioPHP: BioPHP. http://biophp.org (2020). Accessed 10 Aug 2020.

13. Millman KJ, Aivazis M. Python for Scientists and Engineers. *Comput Sci Eng*. 2011; doi: 10.1109/MCSE.2011.36.

14. Cass S: The Top Programming Languages 2019. https://spectrum.ieee.org/computing/software/the-top-programming-languages-2019 (2019). Accessed 10 Aug 2020.

15. Cock PJA, Antao T, Chang JT, Chapman BA, Cox CJ, Dalke A, et al.. Biopython: freely available Python tools for computational molecular biology and bioinformatics. *Bioinformatics*. 2009; doi: 10.1093/bioinformatics/btp163.

16. Django: Django The web framework for perfectionists with deadlines. https://www.djangoproject.com (2020). Accessed 10 Aug 2020.

17. Eilbeck K, Lewis SE, Mungall CJ, Yandell M, Stein L, Durbin R, et al.. The Sequence Ontology: a tool for the unification of genome annotations. *Genome Biol*. 2005; doi: 10.1186/gb-2005-6-5-r44.

18. Gene Ontology Consortium. The Gene Ontology (GO) database and informatics resource. *Nucleic Acids Res*. 2004; doi: 10.1093/nar/gkh036.

19. Machado: Machado - Demonstration Website. https://www.machado.cnptia.embrapa.br (2020). Accessed 10 Aug 2020.

20. PlantAnnot: Plant Co-expression Annotation Resource. https://www.machado.cnptia.embrapa.br/plantannot (2020). Accessed 10 Aug 2020.

21. Machado: Machado Documentation. https://machado.readthedocs.io (2020). Accessed 10 Aug 2020.

22. Machado: Machado Docker. https://github.com/lmb-embrapa/machado-docker (2020). Accessed 10 Aug 2020.

23. Phytozome: Phytozome. https://phytozome.jgi.doe.gov (2020). Accessed 10 Aug 2020.

24. Mudadu MA; Zerlotini A: Supporting data for "Machado: open source genomics data integration framework" GigaScience Database. 2020. http://dx.doi.org/10.5524/100785.

Figure 1

Click here to access/download;Figure;figure1-homeSearchPages.png

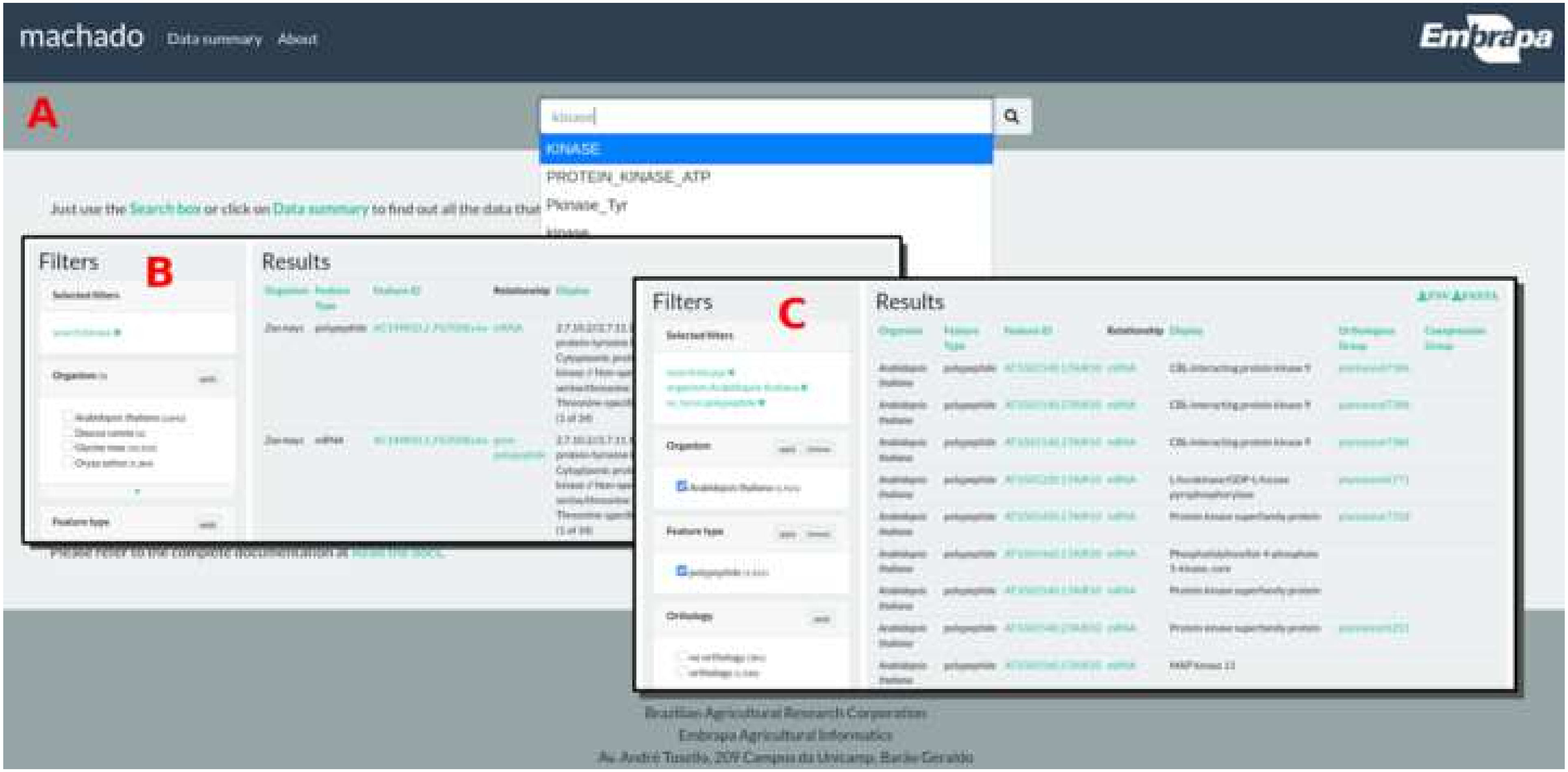

Figure 2

Click here to access/download;Figure;figure2-featurePage.png

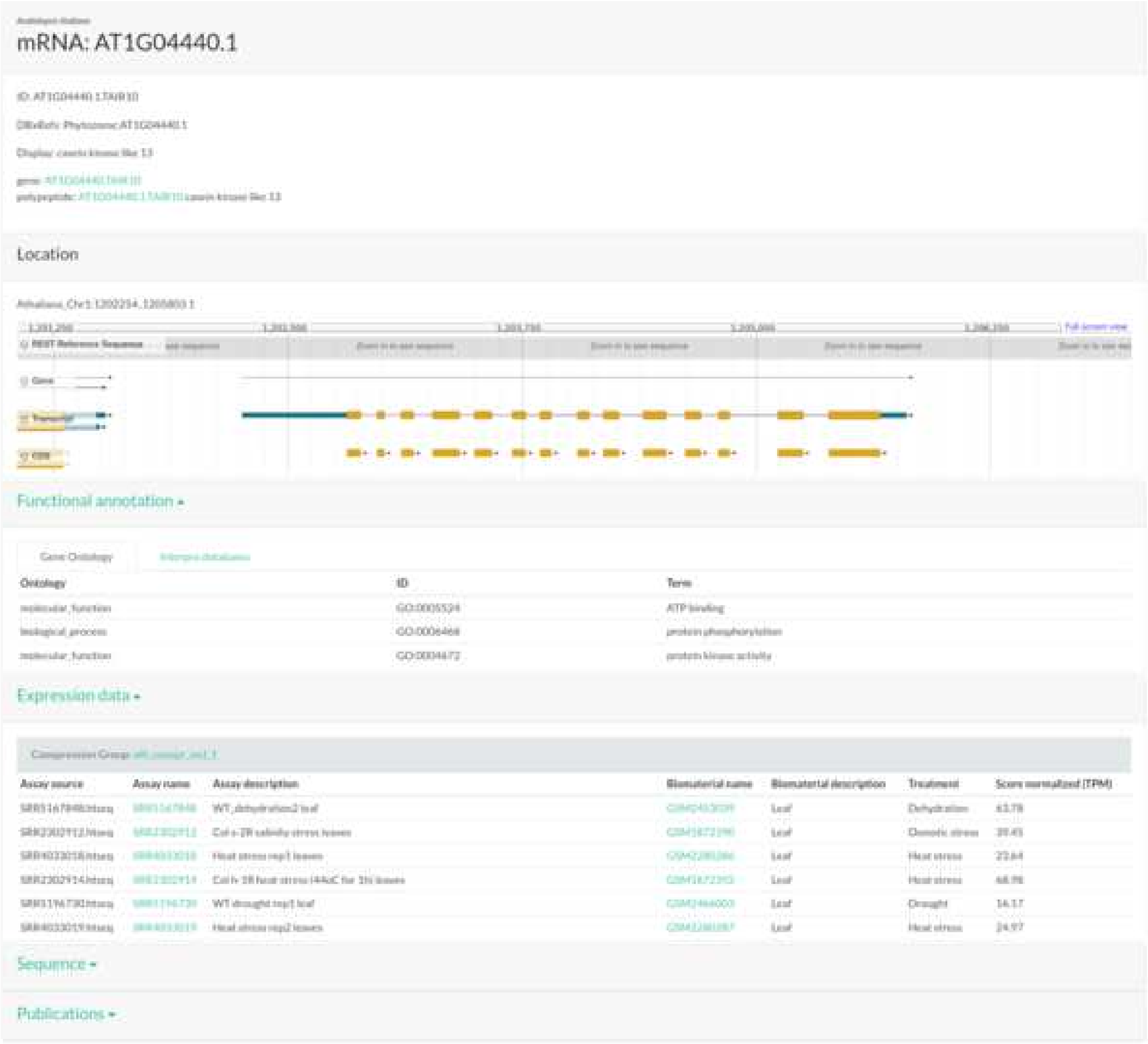

Supplement: giaa097_GIGA-D-20-00137_Revision_3 [file giaa097_giga-d-20-00137_revision_3.pdf]
