# Supplementary material for: Machado: Open source genomics data integration framework
Source: Gigascience. 2020 Sep 14;9(9):giaa097. doi: 10.1093/gigascience/giaa097 (PMC7490629; doi:10.1093/gigascience/giaa097)
Supplement: giaa097_Response_to_Reviewer_Comments_Revision_1 [file giaa097_response_to_reviewer_comments_revision_1.pdf]

Author's Response To Reviewer Comments

Close

Reviewer 1

1) How can new analysis tools be integrated in this framework? Multi-omics data analysis is a more critical issue than data storing or visualization. What kinds of data analytics tool will be available? How to provide the flexibility to integrate third-party software in this framework?

Currently, there are several multi-omics data sets being generated in our institution and it's a major issue Machado intended to deal with primarily. We decided that any multi-omics data analysis should initially be processed separately and the results loaded to the database. This enables users to select features based on the analysis results without having to wait for the analysis to run, which makes Machado faster and simpler. However, this can change in the future. It it possible to integrate analysis tools to Machado as it provides direct access to features, sequences and analysis results via API. There's much to improve in the API interface and we're working on it regularly. Developers can also access the data via the object-relational model or the ElasticSearch index.

2) For the data integration, how can external database be integrated? The framework provides the flexibility to link with other databases? Databases does not only mean relational databases, but can be multiple data sources or biological databases such as KEGG.

The current version of Machado already contains links to other databases such as the NCBI SRA and GEO databases. It's quite simple to add links to external databases by editing the feature template. Each feature may have several dbxrefs (DB external reference) that enable to directly link to external data sources. The integration with different data sources, such as KEGG, is not complicated. Our objective is really to expand these data sources in order to integrate as much information as possible.

3) What kinds of benefit does the function of search powered by ElasticSearch engine provide to users? Seems all components already exist and this proposed framework just implemented by integrating them.

The search engines available in other open source frameworks to integrate multi-omics data are split in several search boxes for different parameters. For example: ID search, GO term search, Gene name search, Organism search, and so on. The user have to execute each search separately and then run another tool to intersect the searches results. The benefit of using the ElasticSearch is to execute complex queries using a single search box, such as, 'calcium GO:0016020 PF02705', which will select features that transport (PF02705) calcium through the membrane (GO:0016020). The result of this query can be additionally filtered by several criteria, such as, organism, expression, orthology, and so on. Although it's true that "components already exist and the framework just implemented it", the novelty is that Machado provides the software to generate and access the ElasticSearch index automatically from a Chado database.

Reviewer 2

- The export icons for TSV/FASTA are identical. This is a UX issue that should be addressed.

The export icons were updated.

- To facilitate deployment/usage, it may be beneficial to create a Dockerfile that can read a configuration and allow users to immediately test Machado on their existing Chado database.

The docker image for Machado is available at https://github.com/lmb-embrapa/machado-docker
This repository also contains a docker-composer and sample data in order to have a Machado instance running straightway.

- documentation for the API appears to be lacking. When I go to https://www.machado.cnptia.embrapa.br/demo_machado/api/ I get 'undefined' for some queries, and it is unclear how to use the API.

The Machado API was completely refactored. Now it provides OpenAPI support (https://www.machado.cnptia.embrapa.br/demo_machado/api/?format=openapi) and Swagger documentation (https://www.machado.cnptia.embrapa.br/demo_machado/api/).

- Within the manuscript, there were some minor wording issues the authors should consider revising. For example, it is unclear what a "public wide database" is. There are also a handful of grammatical errors in the manuscript that need to be fixed. Also, the first github link appears to be broken.

The manuscript was revised for grammatical errors. The link was fixed.

Close
