# Supplementary material for: Machado: Open source genomics data integration framework
Source: Gigascience. 2020 Sep 14;9(9):giaa097. doi: 10.1093/gigascience/giaa097 (PMC7490629; doi:10.1093/gigascience/giaa097)
Supplement: giaa097_Reviewer_2_Report_Original_Submission — Daniel Cook -- 6/28/2020 Reviewed [file giaa097_reviewer_2_report_original_submission.pdf]

Reviewer Report

Title: Machado: open source genomics data integration framework

Version: Original Submission **Date:** 6/28/2020

Reviewer name: Daniel Cook

Reviewer Comments to Author:

The authors present Machado, which is a python-based framework for browsing genomic datasets. The software itself is of very high quality. It is version controlled, conforms to formatting standards, is easy to read, is well commented, and critically - it includes excellent documentation. These are attributes common to successful scientific software.

There are some additional features the authors should consider adding.

- The export icons for TSV/FASTA are identical. This is a UX issue that should be addressed.
- To facilitate deployment/usage, it may be beneficial to create a Dockerfile that can read a configuration and allow users to immediately test Machado on their existing Chado database.
- documentation for the API appears to be lacking. When I go to https://www.machado.cnptia.embrapa.br/demo_machado/api/ I get 'undefined' for some queries, and it is unclear how to use the API.

Within the manuscript, there were some minor wording issues the authors should consider revising. For example, it is unclear what a "public wide database" is. There are also a handful of grammatical errors in the manuscript that need to be fixed. Also, the first github link appears to be broken.

Overall, I believe Machado is of high quality and worthy of publications. The authors should consider revising their manuscript for grammatical errors and consider the software suggestions above.

Level of Interest

Please indicate how interesting you found the manuscript: Choose an item.

Quality of Written English

Please indicate the quality of language in the manuscript: Choose an item.

Declaration of Competing Interests

Please complete a declaration of competing interests, considering the following questions:

- Have you in the past five years received reimbursements, fees, funding, or salary from an organisation that may in any way gain or lose financially from the publication of this manuscript, either now or in the future?

- Do you hold any stocks or shares in an organisation that may in any way gain or lose financially from the publication of this manuscript, either now or in the future?
- Do you hold or are you currently applying for any patents relating to the content of the manuscript?
- Have you received reimbursements, fees, funding, or salary from an organization that holds or has applied for patents relating to the content of the manuscript?
- Do you have any other financial competing interests?
- Do you have any non-financial competing interests in relation to this paper?

If you can answer no to all of the above, write 'I declare that I have no competing interests' below. If your reply is yes to any, please give details below.

I declare that I have no competing interests

I agree to the open peer review policy of the journal. I understand that my name will be included on my report to the authors and, if the manuscript is accepted for publication, my named report including any attachments I upload will be posted on the website along with the authors' responses. I agree for my report to be made available under an Open Access Creative Commons CC-BY license (http://creativecommons.org/licenses/by/4.0/). I understand that any comments which I do not wish to be included in my named report can be included as confidential comments to the editors, which will not be published.

Choose an item.

To further support our reviewers, we have joined with Publons, where you can gain additional credit to further highlight your hard work (see: https://publons.com/journal/530/gigascience). On publication of this paper, your review will be automatically added to Publons, you can then choose whether or not to claim your Publons credit. I understand this statement.

Yes Choose an item.
